# Poster Session II - A263 HIGH RISK OF COLORECTAL CANCER AFTER COLONIC HIGH-GRADE DYSPLASIA IN PATIENTS WITH INFLAMMATORY BOWEL DISEASE

**DOI:** 10.1093/jcag/gwaf042.263

**Published:** 2026-02-13

**Authors:** L M van Lierop, M E Derks, M te Groen, L A Derikx, I D Nagtegaal, F Hoentjen

**Affiliations:** Gastroenterology, University of Alberta Faculty of Medicine & Dentistry, Edmonton, AB, Canada; Radboudumc Afdeling Maag- Darm- en Leverziekten, Nijmegen, GE, Netherlands; Radboudumc Afdeling Maag- Darm- en Leverziekten, Nijmegen, GE, Netherlands; Erasmus MC, Rotterdam, ZH, Netherlands; Radboud universitair medisch centrum, Nijmegen, GE, Netherlands; Gastroenterology, University of Alberta Faculty of Medicine & Dentistry, Edmonton, AB, Canada

## Abstract

**Background:**

There are limited data on colorectal cancer (CRC) risk after a previous diagnosis of high-grade dysplasia (HGD) in inflammatory bowel disease (IBD).

**Aims:**

To determine the long-term CRC risk and cumulative incidence of metachronous colorectal neoplasia (CRN) after a first diagnosis of HGD in IBD, and to assess utilization of HGD treatment strategies over the past three decades.

**Methods:**

In this nationwide retrospective cohort study, data from patients with colonic IBD and a diagnosis of HGD between 1991 and 2021 were extracted from the Dutch nationwide pathology databank (PALGA). The primary outcome was the cumulative incidence of metachronous CRC. Kaplan-Meier curves were used to show proctocolectomy-free survival per decade.

**Results:**

CRC was diagnosed in 348 of 1,220 patients with HGD (28.5%). Of these, 204 patients (16.7%) were diagnosed with CRC within 6 months after the first HGD diagnosis and were considered synchronous cases. Metachronous CRC was diagnosed in 144 of 1,016 patients (14.2%) after a median of 3.6 years. The 1-, 5-, and 10-year cumulative incidences of metachronous CRC after HGD were 2.9%, 10.0%, and 15.9%, respectively. The 1-, 5-, and 10-year cumulative incidences of metachronous CRN were 18.3%, 54.0%, and 75.2%, respectively. We observed a decrease in proctocolectomy-free survival after HGD over time (Table 1).

**Conclusions:**

The combined risk of synchronous and metachronous CRC after a diagnosis of HGD is almost 30%. The advantages of colon-sparing treatment should be balanced with the higher risk of metachronous CRC and the subsequent need for stringent endoscopic surveillance.

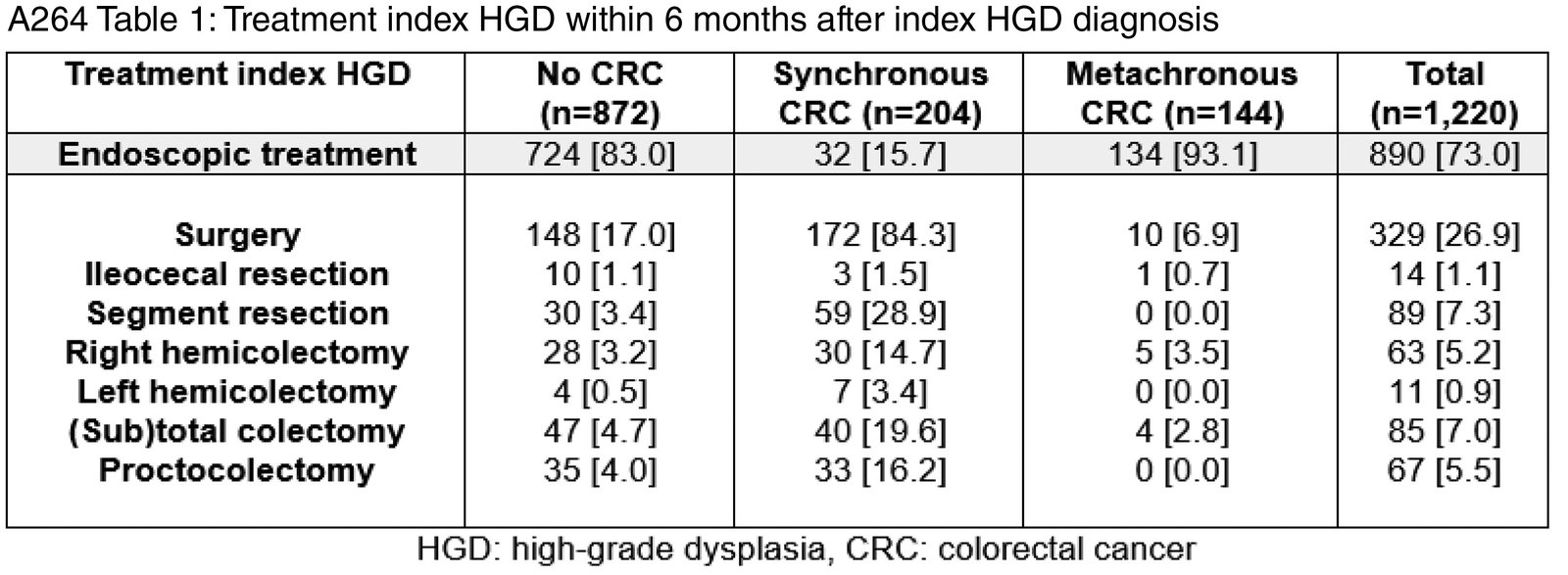

**Funding Agencies:**

None

